# Normalization of microarray expression data using within-pedigree pool and its effect on linkage analysis

**DOI:** 10.1186/1753-6561-1-s1-s152

**Published:** 2007-12-18

**Authors:** Yoonhee Kim, Betty Q Doan, Priya Duggal, Joan E Bailey-Wilson

**Affiliations:** 1 Inherited Disease Research Branch, National Human Genome Research Institute, National Institutes of Health, 333 Cassell Drive, Suite 1200, Baltimore, Maryland 21224, USA; 2 Department of Biostatistics and Epidemiology, School of Public Health, Seoul National University, 28 Yeungundong, Chongnogu, Seoul 110-799, Republic of Korea; 3 McKusick-Nathans Institute of Genetic Medicine, School of Medicine, Johns Hopkins University, 733 North Broadway, Baltimore, Maryland 21205, USA

## Abstract

"Genetical genomics", the study of natural genetic variation combining data from genetic marker-based studies with gene expression analyses, has exploded with the recent development of advanced microarray technologies. To account for systematic variation known to exist in microarray data, it is critical to properly normalize gene expression traits before performing genetic linkage analyses. However, imposing equal means and variances across pedigrees can over-correct for the true biological variation by ignoring familial correlations in expression values. We applied the robust multiarray average (RMA) method to gene expression trait data from 14 Centre d'Etude du Polymorphisme Humain (CEPH) Utah pedigrees provided by GAW15 (Genetic Analysis Workshop 15). We compared the RMA normalization method using within-pedigree pools to RMA normalization using all individuals in a single pool, which ignores pedigree membership, and investigated the effects of these different methods on 18 gene expression traits previously found to be linked to regions containing the corresponding structural locus. Familial correlation coefficients of the expressed traits were stronger when traits were normalized within pedigrees. Surprisingly, the linkage plots for these traits were similar, suggesting that although heritability increases when traits are normalized within pedigrees, the strength of linkage evidence does not necessarily change substantially.

## Background

Genetical genomics [1] integrates genome-wide expression profile data of microarray experiments and marker-based measures of genetic variation. It has newly become a central methodology in quantitative trait studies in order to determine loci involved in regulatory expression of quantitative variation in RNA level. As a result of the reduced cost of microarray expression arrays and marker genotyping, these studies have been extended to study quantitative traits in linkage and association studies [2,3]. In microarray experiments, research on determining which normalization method best adjusts for systematic experimental variation to produce unbiased data is necessary. Among the normalization methods used for the common Affymetrix GeneChip, the robust multiarray average (RMA) method and the statistical algorithm implemented in Affymetrix's Microarray Suite (MAS5) program are the gold standard to control for systematic variation in samples of unrelated individuals [4,5]. Investigation of the effects of various normalization methods in family data are needed [6].

**Figure 1:**
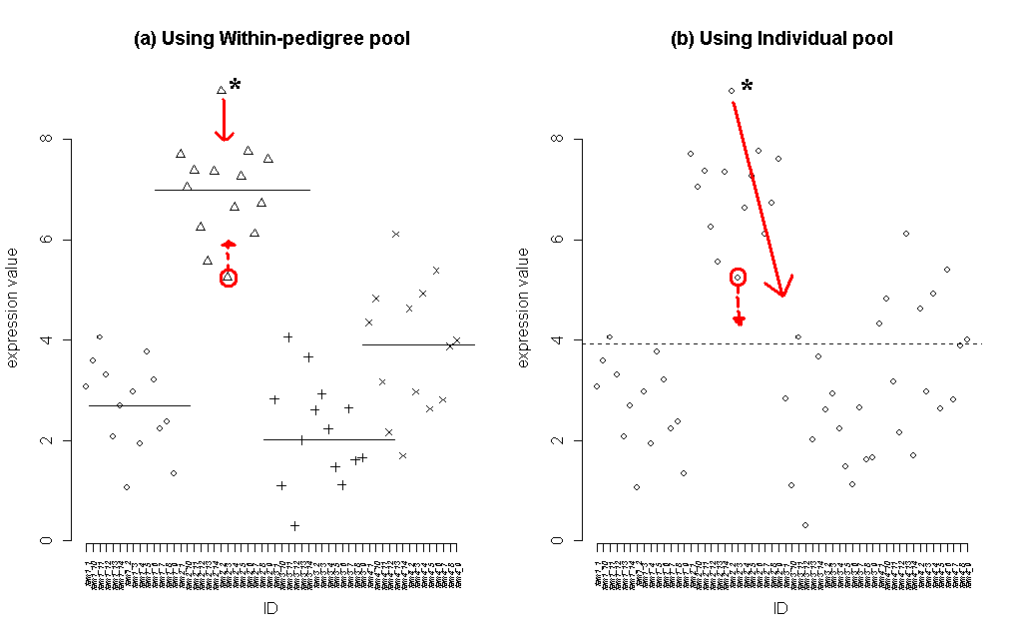
**Adjustment of systematic variation by normalization using within-pedigree pool and using all individuals pool**. a, RMA using a within-pedigree pool: an outlier with systematic variation will be adjusted toward the familial mean of that particular gene expression value. Each symbol (circle, triangle, cross, and X) represents the family and horizontal lines indicate the familial mean value. b, RMA using all individuals as the pool (all subjects shown as circles): an outlier will be adjusted toward the mean of all individuals' gene expression values.

RMA adjusts for systematic variation by performing a quantile normalization procedure, which assumes that the data for the variable considered (such as study sample) all are sampled from the same or similar distributions and the values for that variable are then normalized to a standard distribution. However, it is not yet known which standard distribution is the best to use for family data in genetical genomics studies. Linkage analysis utilizes data from pedigrees, which usually have more homogeneity within pedigrees for the trait under study than between pedigrees, both biologically and environmentally. Thus, the increased background sharing within pedigrees can result in increased correlation between related individuals for expression levels. Consequently, the familial correlation within each pedigree due to biological similarity is of interest in linkage studies. Therefore, we hypothesized that linkage analysis that uses expression trait data normalized by ignoring pedigree membership could improperly 'correct' the trait values by imposing equal means and variances across pedigrees. A conceptual graph (Fig. 1) plots the values of one trait for each individual. It shows how an outlier in a family, whose members have a different distribution of expression values than other families, will be systematically adjusted (solid arrow) toward the familial mean value if its value is normalized by within-pedigree normalization methods. However, the normalized trait values will be over-corrected and biased toward the overall mean if normalized using all individuals because this method falsely assumes that they are unrelated individuals sampled from a single distribution. A second member of the same family would be adjusted in a different direction (dashed arrow) away from the overall mean and toward the family mean if its value is normalized by within-pedigree normalization.

Our aim is to maintain the individual familial distributions with normalization. We address this problem of normalization of expression data within pedigrees by comparing two possible standard distributions for normalization: 1) applying RMA across all arrays as a pool, which assumes all individuals are independent and share the same distribution of trait values, and 2) applying RMA to arrays within pedigrees assuming family members within a pedigree share the same distribution.

## Methods

Study subjects consisted of 194 individuals from 14 Centre d'Etude du Polymorphisme Humain (CEPH) Utah pedigrees, and 2882 autosomal and X-linked single-nucleotide polymorphism (SNP) genotypes were available from The SNP Consortium [2]. Quantitative phenotype data were generated from immortalized B cells, and 8793 gene expression values were available from the microarray raw CEL (cell intensity file) data files from the Affymetrix Genechips and Hgfocus CDF (chip description file) files. In cases in which a subject had more than one expression file, the first replicate was chosen for this analysis so that we used one array per subject. To evaluate the performance of different scenarios, two data sets were used: 1) RMA normalized using all individuals (arrays) as the normalization pool, and 2) RMA normalization applied to a within-pedigree pool to be able to allow for as many distributions as the number of pedigrees. The 'affy' package v1.8 in R v2.3.1 was used to perform the normalizations of the expression data [7].

In order to examine the effect of using different normalization pools consisting of different types of individuals, we performed paired *t*-tests using 8793 gene expression values of four founders of one family in two ways: comparing the values of the four founders after normalizing using themselves as the pool to paired gene expression values after normalizing 1) using all family members including themselves as the pool, and 2) using independent individuals as the pool (other grandparents from other families) [8]. The purpose of microarray normalization methods is to remove systematic variation while preserving biological variation. Therefore, comparison of the normalized gene expression data using the four founders as their own normalization pool to the normalized data using either of the other two pools (family members or independent individuals) should not show significant differences if the normalization pools are all only removing random error variation. Thus, we should observe non-significant *p*-values for these paired *t*-tests if only random variation is removed by each method. We assumed that these *t*-tests indicated a significant difference in the normalization methods if the *p*-value was less than a conservative *p*-value of 0.001 (since we were performing over 8,000 tests). However, we also evaluated this using a *p*-value of 0.05 as the significance threshold.

To examine the effect of normalization methods on linkage results, we selected 18 *cis*-acting transcriptional regulator phenotypes with previous evidence of *cis*-acting linkage to the known location of each corresponding structural gene [2]. Nonparametric quantitative linkage analysis was performed using Merlin v1.0 with the qtl option. We plotted both the negative *p*-values of the nonparametric linkage score [8] and the allele-sharing LOD score of Kong and Cox [9]. Based on the change of mean and variance of the trait values in each array, some individuals may have different trait values when using different normalization methods. FCOR in S.A.G.E v5.1.0 was used to calculate familial correlations (e.g., parent-offspring, sibling, and grandparent), which were compared for each trait across the different normalization methods.

## Results and discussion

**Figure 2:**
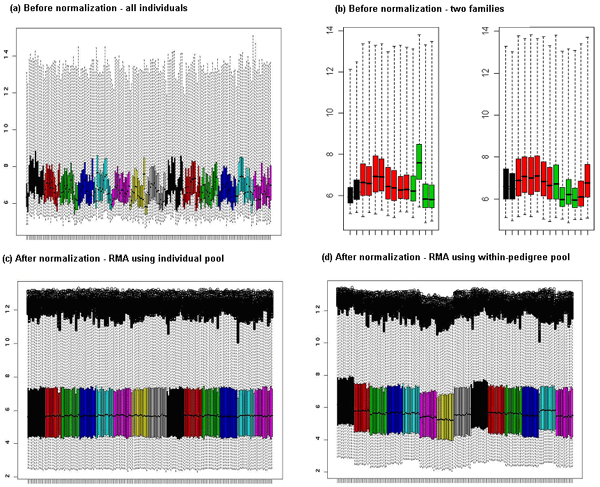
**Box plots of 194 individuals across 8793 genes before and after normalization**. a, Box plots of 194 individuals across 8793 genes before normalization. b, Box plots from the representative two families before normalization. Green, grandparents; black, parents; red, siblings. c, After RMA normalization using all individuals (arrays) as normalization pool. d, After RMA normalization using within-pedigree pool. For panels a, c, and d, each color represents a different family.

When comparing the gene expression data normalized using four individuals as their own normalization pool versus using their family members as a pool, 39% of traits had a *p*-value < 0.001 and 91% had a *p*-value < 0.05. Interestingly, 95% of the genes had a *p*-value < 0.001 and 99.5% had a *p*-value < 0.05 when comparing data normalized using the four individuals as their own normalization pool versus using independent individuals as the normalization pool. This suggests that using independent individuals as the normalization pool may be removing biological variation in addition to removing random error. Figure 2a shows the different means and variances individually before normalization colored distinctively by family. Prior to normalization, the pedigrees appear to have familial trends, with siblings having similar trait values while unrelated grandparents are more different (Fig. 2b). These might reflect the potential genetic correlations in expression values within pedigrees. After normalization using all individuals as the normalization pool (Fig. 2c), all subjects were aligned at the overall mean and variance. However, when the normalization uses the within-pedigree pool, the mean and variance were aligned distinctly for each family (Fig. 2d). Plots of the within-pair trait differences for the two normalization methods show that some sib-pairs had very similar values across these methods, whereas there was a marked change in these within-pair trait difference values for other sib pairs (data not shown).

**Figure 3:**
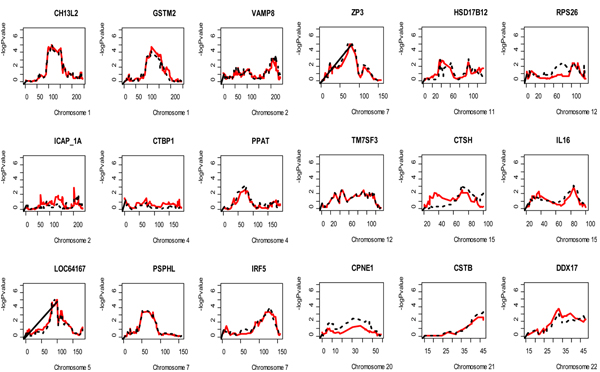
**Results of nonparametric quantitative linkage analysis of 18 *cis*-acting gene expression values using Merlin**. Plots of negative log p-values (Y axis) of 18 *cis*-acting genes in nonparametric linkage analysis. Red solid line: LOD score using phenotypes normalized by RMA using a within-pedigree pool. Black dashed line: LOD score using phenotypes normalized by RMA using all individuals as a normalization pool.

In Table 1, we show that the familial correlation coefficients of expression phenotype values have changed according to the normalization pool. Generally, the correlation coefficients between all relative pairs are higher in the data normalized within pedigrees than in the data after normalization using all individuals. *CPNE1*, *CSTB*, *ICAP_1A*, and *TM7SF3 *have negative familial correlation coefficients (bold in Table 1) when they are calculated with the phenotypes after normalizing using all individuals as the normalization pool. Those coefficients are positive after normalizing using the within-pedigree pool. This suggests that the within-pedigree method preserves the underlying biological variation patterns while removing the systematic variation, since we have strong prior evidence that 18 *cis*-acting genes are involved in the regulation of these gene expression levels. The results of nonparametric quantitative linkage show that normalization using either the all-individuals pool or the within-pedigree pool yields similar maximum allele-sharing LOD scores for signals within 20 cM of the target structural gene. However, most of these allele-sharing LOD scores were slightly decreased for the within-pedigree pool compared to the individual pool (15/18). The difference in maximum LOD scores between the methods ranged from 0.7 to 0.01. When we compared the negative log *p*-values of the NPL scores, the patterns of most genes normalized within-pedigree (red solid line) and using all individuals in the normalization pool (black dashed line) were similar except for *RPS26 *and *CTSH *(Fig. 3).

**Table 1:** Familial correlation coefficients and maximum LOD score of non-parametric linkage analysis after RMA normalization

		Familial correlation coefficient^a^							
				
		Parent-offspring		Sibling		Grandparent-grandchild		Maximum LOD^b^	
					
Gene	Target gene loci	All arrays pool	Within pedigree pool	All arrays pool	Within-pedigree pool	All arrays pool	Within-pedigree pool	All arrays pool	Within-pedigree pool
*CHI3L2*	1p12	0.308	0.2689	0.1477	0.1146	0.1274	0.082	4.08	4.25
*GSTM2*	1p13	0.2887	0.3217	0.3284	0.3941	0.2277	0.3412	3.15	3.58
*VAMP8*	2p11	0.0939	0.4196	0.4304	0.7284	0.0475	0.4261	2.7	2.18
*ICAP_1A*	2p25	**-0.2898**^c^	**0.1242**	0.4277	0.7435	**-0.1441**	**0.5436**	1.23	1.97
*CTBP1*	4p16	0.3012	0.3772	0.5581	0.639	0.2002	0.3534	0.81	0.58
*PPAT*	4q12	0.2055	0.2545	0.4469	0.4539	0.0778	0.1774	2.24	1.77
*LOC64167*	5q15	0.2927	0.3464	0.2254	0.2843	0.0483	0.1295	4.61	4.12
*PSPHL*	7p11	0.3932	0.5599	0.4086	0.5207	0.223	0.4089	2.51	2.54
*ZP3*	7q11	0.4084	0.6565	0.43	0.7211	0.2311	0.5527	4.61	3.93
*IRF5*	7q32	0.3462	0.384	0.2874	0.314	0.0825	0.1731	3.06	2.42
*HSD17B12*	11p11	0.2375	0.7565	0.3892	0.8512	0.1205	0.7026	2.06	1.96
*TM7SF3*	12p11	0.0618	0.5035	0.1892	0.6145	**-0.1298**	**0.3133**	1.67	1.41
*RPS26*	12q13	0.5907	0.6533	0.7628	0.8023	0.3606	0.453	1.58	1.57
*CTSH*	15q25	0.421	0.5694	0.5442	0.6546	0.322	0.5205	2.29	1.6
*IL16*	15q26	0.0235	0.1814	0.4244	0.5689	0.047	0.2333	2.18	1.97
*CPNE1*	20q11	**-0.0113**	**0.3377**	0.3958	0.73	0.0512	0.3515	1.52	0.68
*CSTB*	21q22	0.1592	0.3987	0.2717	0.5898	**-0.0075**	**0.2784**	2.31	1.69
*DDX17*	22q13	0.1687	0.2588	0.1911	0.5493	0.1911	0.2523	2.02	2.74

^a^Correlation coefficients of 18 gene expression values by familial pairs using FCOR, S.A.G.E.v5.1.0.

^b^Maximum allele-sharing LOD score of nonparametric linkage analysis after RMA normalization.

^c^Bold text indicates negative familial correlation coefficient.

## Conclusion

Because of the presence of systematic variation in generating gene expression data, proper normalization of raw values is required to separate true signals from background noise. Previous gene expression studies have typically focused on unrelated individuals. With the emergence of genetic linkage studies of gene expression traits, the use of pedigrees poses concerns for proper normalization. We investigated the need to account for pedigrees by normalizing within families. Our results for the 18 linked traits show strong familial correlations, which generally increased when traits were normalized within pedigrees. However, in general, the strength of the maximum LOD score decreased (13/18) when traits were normalized within pedigrees. This is not surprising because the increased correlation of a quantitative trait within a family may decrease evidence for linkage. It is also possible that linkage signals are inflated when normalizing using all individuals as the pool. Because we did not evaluate linkage of these 18 traits to all markers on other chromosomes in this data set, we cannot evaluate possible inflation of linkage signals. However, the pattern of the chromosomal linkage plot for these genes (15/18) did not differ across the two normalization strategies. This lack of difference suggests that normalization within pedigrees may not be necessary, at least in studies with small pedigree sizes. However future linkage studies on expression data with larger pedigree sizes and/or large sample sizes may benefit from normalization by pedigree.

## Competing interests

The author(s) declare that they have no competing interests.
